# CDK1 interacts with iASPP to regulate colorectal cancer cell proliferation through p53 pathway

**DOI:** 10.18632/oncotarget.17794

**Published:** 2017-05-11

**Authors:** Wei Gan, Hua Zhao, Tiegang Li, Kuijie Liu, Jiangsheng Huang

**Affiliations:** ^1^ Department of General Surgery, the Second Xiangya Hospital, Central South University, Changsha 410011, Hunan, China; ^2^ Department of Minimally Invasive Surgery, the Second Xiangya Hospital, Central South University, Changsha 410011, Hunan, China

**Keywords:** CDK1, iASPP, colorectal cancer, cell proliferation, p53

## Abstract

CDK1 (cyclin-dependent kinase 1) is a critical regulator of the G2-M checkpoint. CDK1 is considered a possible target for cancer treatment. In addition to CDK1, iASPP plays essential role in maintaining cancer cell proliferation. In the present study, we monitored the expression of CDK1 and iASPP at mRNA and protein levels in CRC tissues and cell lines; we also predicted that iASPP protein might interact with CDK1 protein. By performing GST pull-down assay and Co-IP assay, we confirmed the interaction of CDK1 and iASPP protein. In CRC cell lines, CDK1 interacted with iASPP to affect CRC cell proliferation and apoptosis; moreover, the p53 apoptosis pathway was involved in this progression. Taken together, we revealed that CDK1 and iASPP was up-regulated in CRC tissues and cell lines; CDK1 protein interacted with iASPP protein to affect CRC cell proliferation and apoptosis through the p53 apoptosis pathway. CDK1 and iASPP might serve as not only promising targets in CRC treatment, but also efficient prognostic markers. From the perspective of protein interactions, we provided a novel theoretical basis for targeted therapy of CRC.

## INTRODUCTION

Colorectal cancer (CRC), one of the most fatal diseased all over the world, has caused enormous economic and spiritual losses to people; identifying reliable prognostic markers and personalized treatment regimen still remain important challenges [1]. The main causes of the poor clinical outcomes in patients with CRC have recently been attributed to deeper tumor location, advanced stage and cancer metastasis at diagnosis, which result in dramatically poorer survival in patients with CRC [2]. Cyclin-dependent kinase 1 (CDK1), a key player which promotes G2-M transition, regulates G1 progress and G1-S transition through associating with multiple interphase cyclins, has been reported to be upregulated in CRC circulating tumor cells [3]. In addition, a high ratio of CDK1 expression of nuclear/cytoplasmic predicts a poorer prognosis in patients with CRC [4]. This inspired us to investigate the role of CDK1 in the regulation of CRC cell proliferation and clinical features in patients with CRC.

Molecules including CDKs which are related to regulation of cell cycle regulation have become research focus and been regarded as potential prognostic and therapeutic markers in diverse cancers [5, 6]. CDKs expression changes in cells often result in abnormal proliferation, as well as dysfunction of related genes or proteins [6]. Cell cycle could be induced to arrest at G1 phase through upregulation of CDK inhibitors, p27KIP1 and p21WAF1, and subsequent downregulation of CDK [7]. CDK1/cyclin B kinase complex, a major regulatory factor of the G2/M transition, has also been regarded as a major regulator of promoting cell cycle transitions at late G2 and mitosis through substrates phosphorylation and activating reorganization of the nuclear envelope, spindle apparatus and actin cytoskeleton. Recently, a study of mouse gene revealed that CDK1 alone is sufficient to trigger and promote the cell cycle of almost all types of cells, indicating its primary role in regulation of cell proliferation [6, 8]. However, in circumstance of cancers, dysfunction of cell cycle regulatory proteins occurs frequently.

Inhibitor of apoptosis stimulating protein of p53 (iASPP), a principle member of the ASPP family, has been regarded as an inhibitor of p53 which is evolutionarily conserved. iASPP is a binding partner of p65 (RelA); it inversely regulates the expression of RelA subunit (p65) of the nuclear factor-kappa B (NF-*κ*B), which has been regarded as an essential factor in the inflammatory response, as well as apoptosis [9, 10]. Given this, it is possible that iASPP expression or iASPP activity would exert influence over NF-*κ*B function, and thus make contribution to cell growth. In the circumstance of cancers, iASPP has been reported to serve as an independent prognostic marker. U251 cell proliferation and growth showed to be significantly suppressed after knockdown of iASPP [11]. In addition, a higher expression of iASPP has been reported in prostate cancer tissues, compared with that of the normal tissues [12]. After shRNA-induced iASPP knockdown, the proliferation of prostate cancer cell showed to be inhibited, whereas the apoptosis of p53-defective prostate cancer cell showed to be promoted. iASPP has also been indicated to participate in tumor metastasis, suggesting its potential role of being an efficient therapeutic target [13, 14].

Moreover, Lu et al. [15] reported the phosphorylation of iASPP by cyclin B1/CDK1 could result in the inhibition of iASPP dimerization and the promotion of iASPP monomer nuclear entry, thus follows the exposure of its p53 binding sites, finally leads to an increased inhibition of p53. In most melanoma cell lines, wild-type p53-expression is usually accompanied with upregulated expression of phosphorylated nuclear iASPP. Suppression of iASPP phosphorylation lead to p53-dependent apoptosis and inhibition on cell proliferation [15].

Given all this, we speculated that CDK1 might interact with iASPP in CRC to modify CRC cell proliferation through the p53 pathway, thus affect the progression of CRC. To validate this speculation, we monitored the expression levels of CDK1 and iASPP in tissues derived from CRC patients at different stages, and different CRC cell lines; tested and verified the interaction between CDK1 and iASPP proteins; finally investigated the functional role of CDK1 and iASPP in regulating CRC cell proliferation through the p53 pathway, and in the prognosis of CRC patients. From the perspective of protein interactions, we provided a novel theoretical basis for targeted therapy of CRC.

## RESULTS

### Expression levels of CDK1 and iASPP and their correlation in CRC tissues

**Figure 1:**
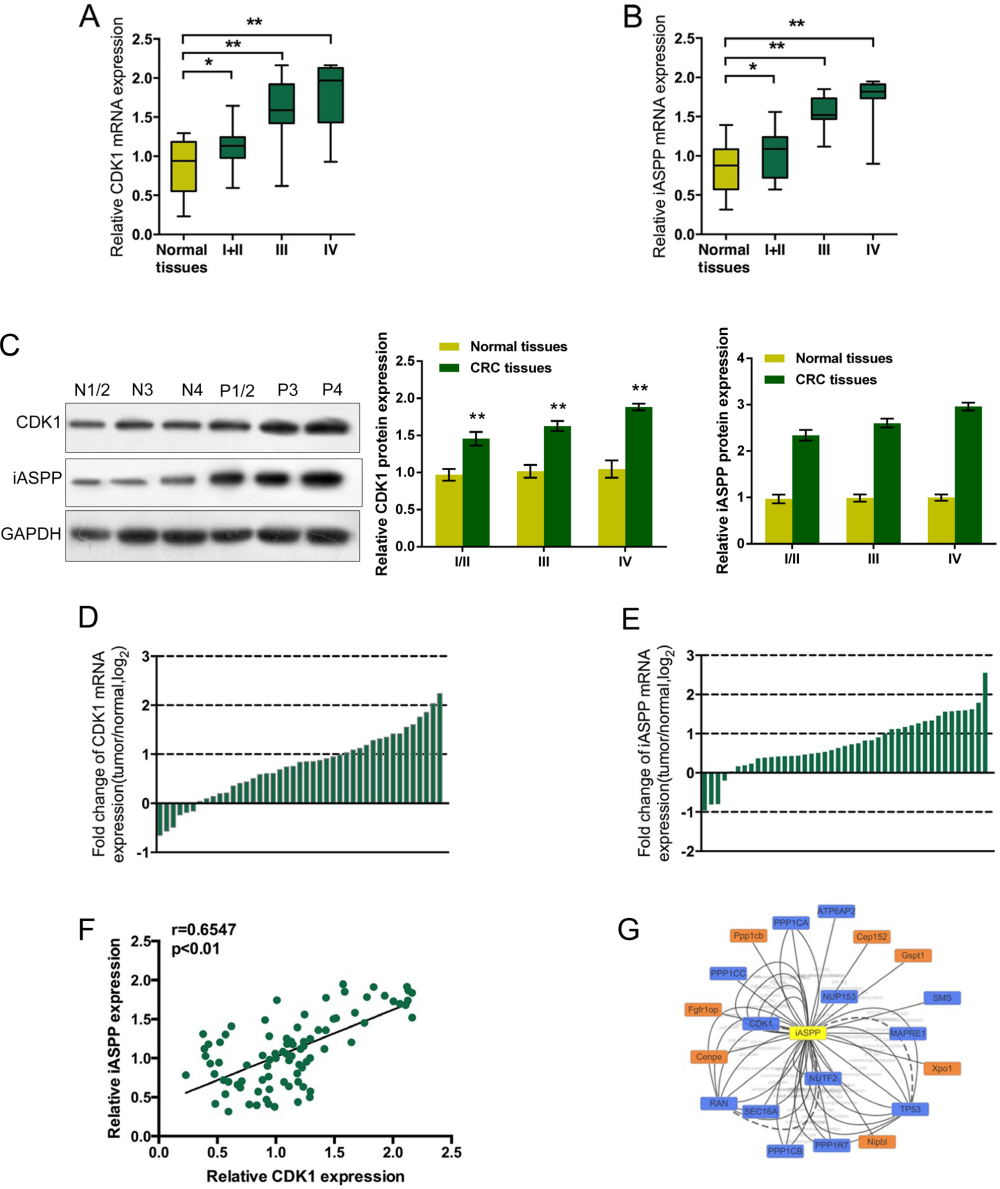
Expression levels of CDK1 and iASPP and their correlation in CRC tissues (**A**) and (**B**) The expression levels of CDK1 and iASPP mRNA in CRC tissues derived from patients in different stages were deteremined by using real-time PCR assays, compared to normal tissues. The data are presented as mean ± SD of three independent experiments. *P < 0.05, **P < 0.01. (**C**) The protein levels of CDK1 and iASPP in tumor tissues and adjacent normal tissues (normal tissues N1/2, N3 and N4; tumor tissues P1/2, P3 and P4) were determined using Western blot assays. (**D**) and (**E**) Expression of CDK1 and iASPP mRNA in 43 pairs of CRC tissues and their corresponding adjacent non-tumorous tissues (ANTs) in a training cohort. Expression level of CDK1 and iASPP mRNA was determined by real-time PCR and normalized to U6. Fold change were analyzed using the formula 2^−(ΔΔCT [CRC/ ANT])^. Red line indicates fold change of CDK1 or iASPP mRNA equal to 2. (**F**) The Spearman's rank correlation analysis was performed to analyze the correlation between CDK1 and iASPP mRNA in CRC tissues. (**G**) Data from Cytoscape showing the candidate proteins which could interact with iASPP protein.

Given the essential roles of CDK1 and iASPP in CRC cell proliferation, we firstly assessed the expression levels of CDK1 mRNA and iASPP mRNA in CRC tissues derived from different stages of CRC patients, compared to matched adjacent normal tissues using real-time PCR assays. Results showed that CDK1 mRNA and iASPP mRNA was highly expressed in CRC tissues, compared to the normal tissues, and higher expressed in tissues derived from patients in advanced stages (Figure 1A and 1B, stage I + II *P* < 0.05, stage III and IV *P* < 0.01.). The protein levels of CDK1 and iASPP in tumor tissues and adjacent normal tissues were determined using Western blot assays. Results showed that CDK1 and iASPP protein levels in tumor tissues were significantly increased, compared to normal tissues (Figure 1C). To validate the data, we performed quantitative real-time PCR in 43 cases of CRC tissues and adjacent normal tissues in training cohort. Compared with the corresponding adjacent normal tissues, CDK1 was significantly up-regulated (more than two-fold [i.e., log2 (fold change) > 1]) in 15 CRC cases (34.88%) (Figure 1D); iASPP was significantly up-regulated (more than two-fold [i.e., log2 (fold change) > 1]) in 15 CRC cases (34.88%) (Figure 1E). By performing the Spearman's rank correlation analysis, we revealed that CDK1 and iASPP expression was positively correlated in CRC tissues (Figure 1F). To search for candidate proteins interacted with iASPP, we performed a Cytoscape analysis. The Cytoscape map showed that iASPP could interact with 20 proteins (Figure 1G), including CDK1. These data suggest that CDK1 and iASPP proteins might interact with each other in CRC to act on CRC cell proliferation.

### Protein levels of CDK1 and iASPP in CRC cell lines

**Figure 2:**
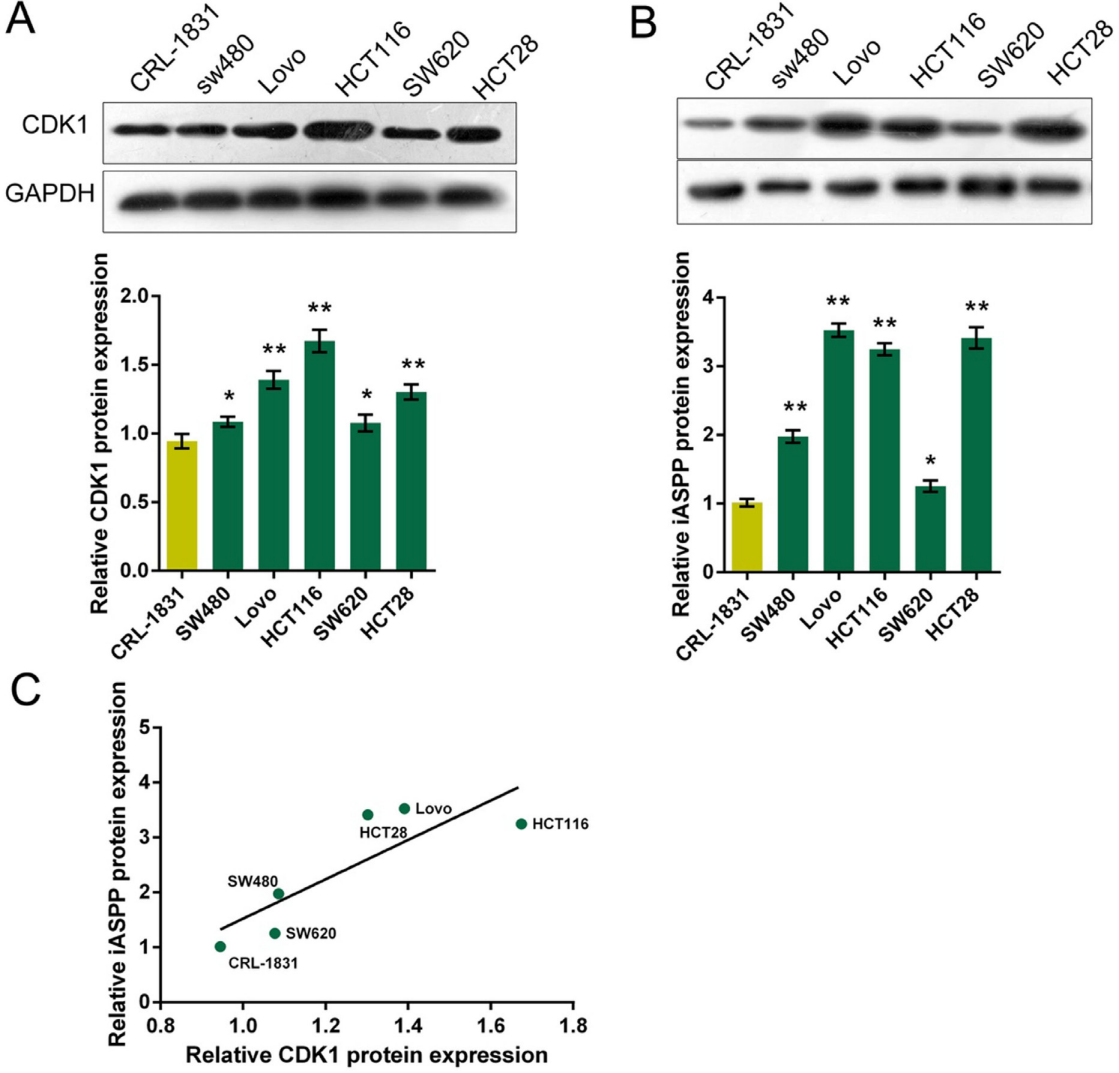
Protein levels of CDK1 and iASPP in CRC cell lines (**A**) and (**B**) The protein levels of CDK1 and iASPP in five CRC cell lines, SW480, LoVo, HCT116, SW620 and HCT28 were determined using Western blot assays, compared to normal cell line, CRL-1831. The data are presented as mean ± SD of three independent experiments. *P < 0.05, **P < 0.01. (**C**) The Spearman's rank correlation analysis was performed to analyze the correlation between CDK1 and iASPP protein in CRC and normal cell lines.

Given that CDK1 mRNA and iASPP mRNA was highly expressed in CRC tissues; we then determined the protein levels of CDK1 and iASPP in five CRC cell lines: HCT116, HCT28, LoVo, SW480 and SW620, compared with a normal cell line, CRL-1831 using Western blot assays. Results showed that CDK1 and iASPP protein levels were promoted in five CRC cell lines, compared to normal cell line (Figure 2A and 2B). The correlation between CDK1 protein and iASPP protein in CRC cell lines was analyzed using the Spearman's rank correlation analysis. Results showed that CDK1 protein and iASPP protein in CRC cell lines was positively correlated (Figure 2C). To see from Figure 2C, CDK1 protein and iASPP protein had the highest correlation in LoVo and HCT116 cells; so we chose LoVo and HCT116 cell lines for tool cells.

### Verification of the interaction between CDK1 and iASPP proteins

**Figure 3:**
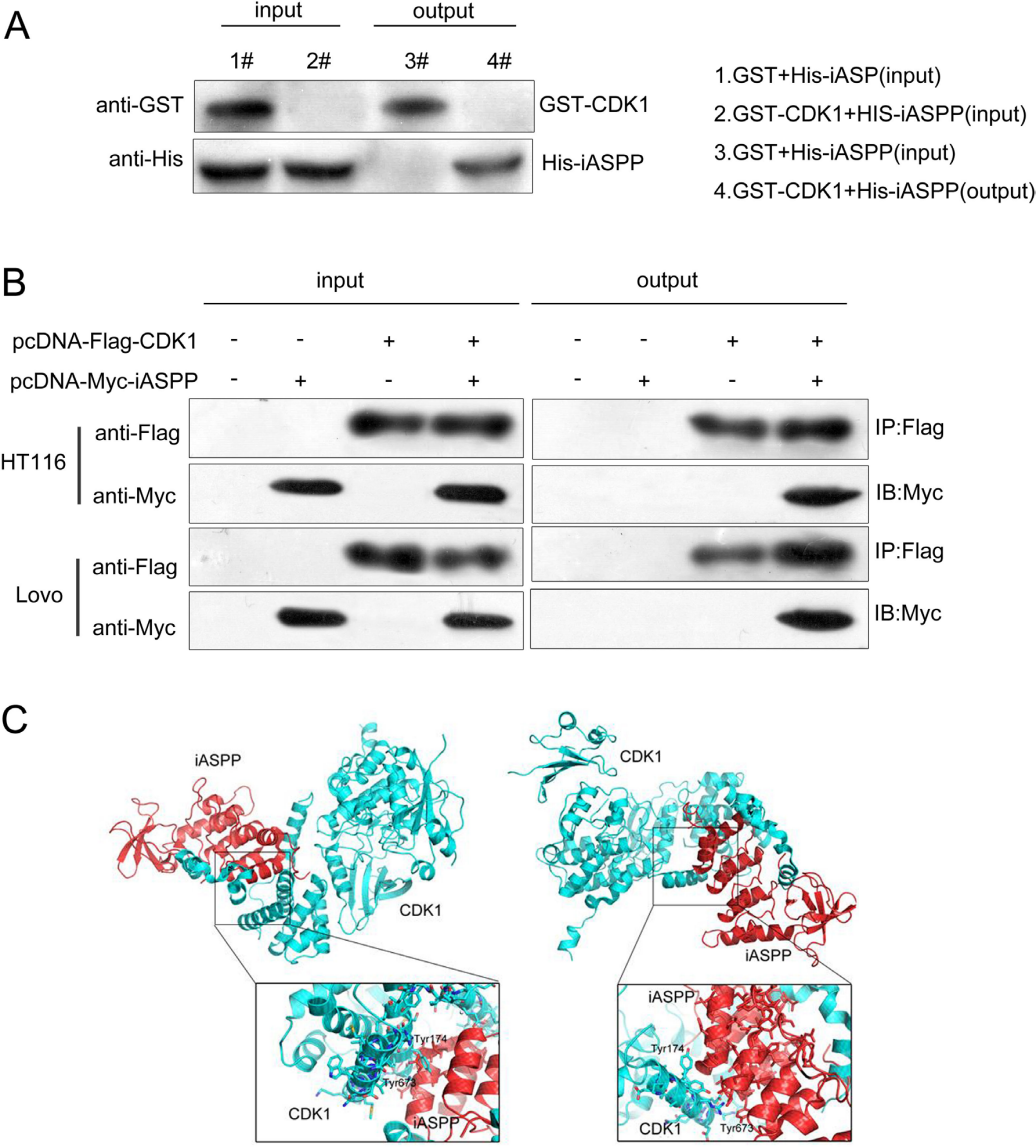
Verification of the interaction between CDK1 and iASPP proteins (**A**) GST pull-down assays showing GST-CKD1 pulled down His-iASPP. (**B**) Confirmation of the interaction between CDK1 and iASPP using Co-IP assays. (**C**) The binding structure schematic diagram showing two possible interacting regions of CDK1 protein and iASPP protein, generated using PyMol.

According to previous studies, iASPP phosphorylation could be induced by cyclin B1/CDK1, which further blocks iASPP dimerization and iASPP monomer nuclear entry, results in the exposure of the p53 binding sites, and finally leads to p53 inhibition [15]. Here we have revealed that CDK1 and iASPP expression was up-regulated in CRC tissues and cell lines; to further verify the interaction between CDK1 and iASPP protein *in vitro* and *in vivo*, GST pull-down assay and Co-IP assay was performed. On GST pull-down assays, GST-tagged CDK1 was used as bait protein and His-tagged iASPP was target protein. The elutes were then analyzed using Western blotting assays. Results showed that His-tagged iASPP protein could be pulled down by GST-tagged CDK1 protein, as shown by Western blot results (Figure 3A), indicating the tight interaction between CDK1 and iASPP proteins. In Co-IP assays, pcDNA-Flag-CDK1 and pcDNA-Myc-iASPP vectors were constructed and co-transfected into HCT116 and LoVo cells. Flag monoclonal antibodies were used for IP testing, followed by Western blot detection using Flag and Myc antibodies. Results from Western blotting showed that CDK1 protein could interact with iASPP protein (Figure 3B). To verify the interaction between CDK1 protein and iASPP protein, the PyMol was used to predict the protein binding structure schematic diagram. As shown in Figure 3C, there might be two independent interacting regions between CDK1 protein and iASPP protein, Tyr174 and Tyr673.

### Effect of co-processing CDK1 knockdown and iASPP overexpression on CRC cell proliferation and apoptosis

**Figure 4:**
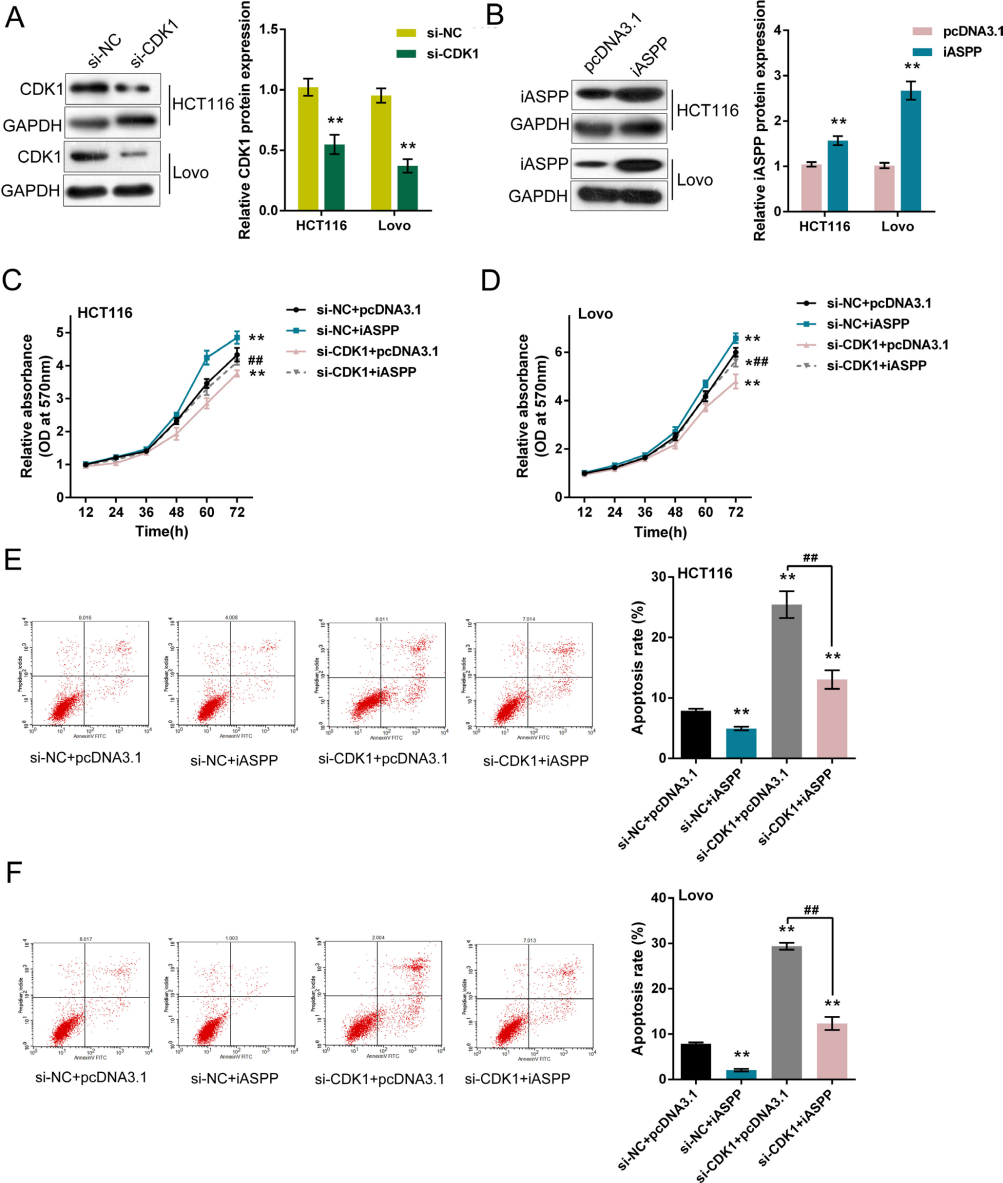
Effect of co-processing CDK1 knockdown and iASPP overexpression on CRC cell proliferation and apoptosis (**A**) si-CDK1 was transfected into HCT116 and LoVo cells to achieve CDK1 knockdown, as verified by Western blot assays. (**B**) pcDNA3.1/iASPP was transfected into HCT116 and LoVo cells to achieve forced iASPP expression, as verified by Western blot assays. (**C**) and (**D**) si-CDK1 and pcDNA3.1/iASPP were co-transfected into HCT116 and LoVo cells; the cell viability was determined using MTT assays. (**E**) and (**F**) si-CDK1 and pcDNA3.1/iASPP were co-transfected into HCT116 and LoVo cells; the cell apoptosis was determined using Flow cytometer assays. The data are presented as mean ± SD of three independent experiments. *P < 0.05, **P < 0.01 (compared to control group), ^##^*P* < 0.01 (compared to si-CDK1 + pcDNA3.1 group.).

We verified the interaction between CDK1 protein and iASPP protein, then we evaluated the effect of co-processing CDK1 knockdown and iASPP overexpression on CRC cell proliferation and apoptosis. CDK1 knockdown was achieved by transfecting si-CDK1 into HCT116 and LoVo cells (Figure 4A), and pcDNA3.1/iASPP was transfected to achieve iASPP overexpression (Figure 4B), as verified using Western blot assays. As exhibited by MTT assays, cell proliferation of HCT116 and LoVo cells was promoted by iASPP overexpression, repressed by CDK1 knockdown; the promotive effect of iASPP on CRC cell proliferation could be partially reversed by CDK1 knockdown (Figure 4C and 4D). Consistent results were observed from the apoptosis assays. Cell apoptosis of HCT116 and LoVo cells were repressed by iASPP overexpression, promoted by CDK1 knockdown; the repressive effect of iASPP on CRC cell apoptosis could be partially reversed by CDK1 knockdown (Figure 4E and 4F). These data suggested that CDK1 protein interacted with iASPP protein in CRC cell lines to affect CRC cell proliferation and apoptosis.

### Requirement of p53 pathway in CRC cell proliferation and apoptosis regulation

**Figure 5:**
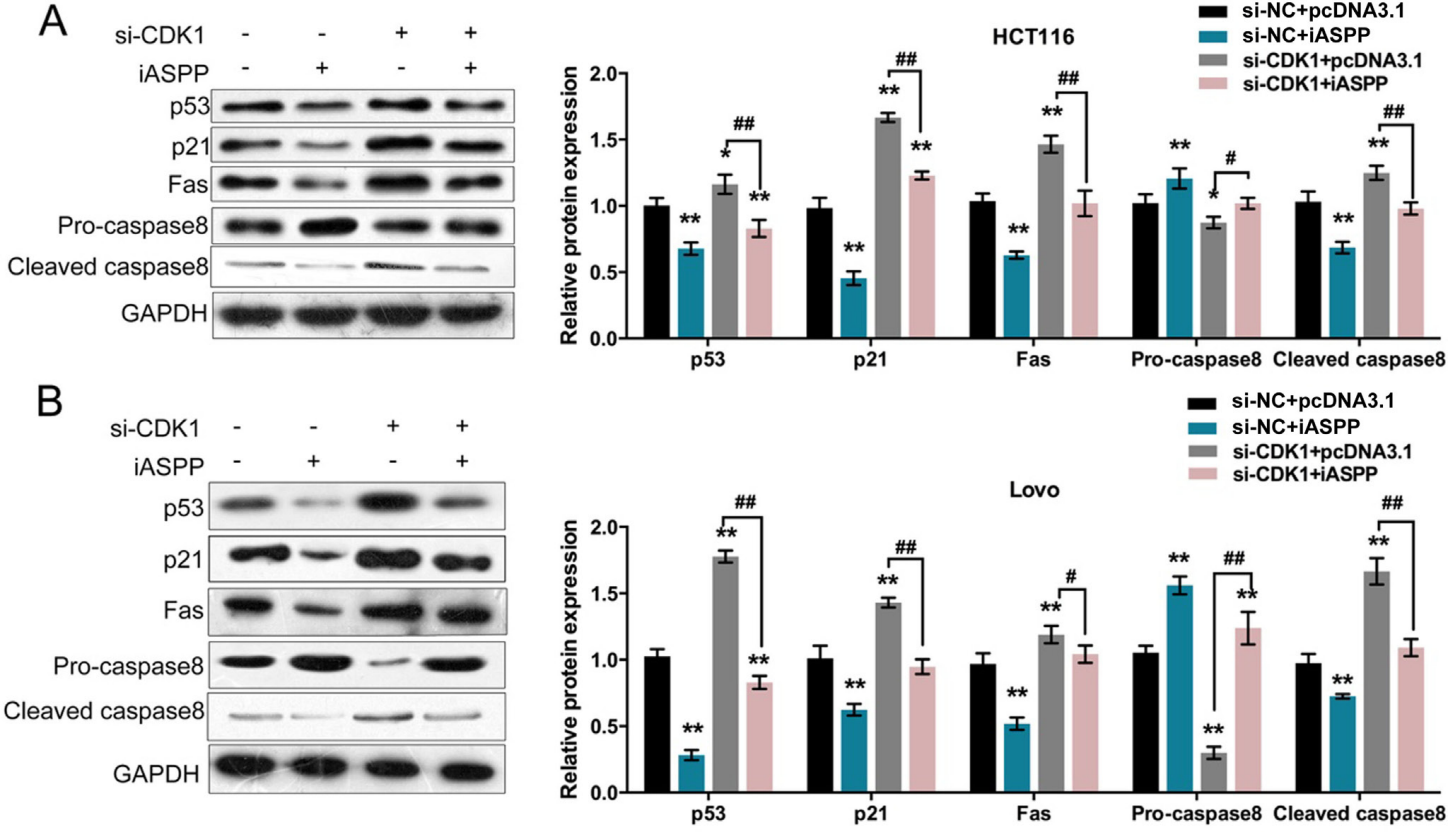
Reqirement of p53 pathway in CRC cell proliferation and apoptosis regulation (**A**) and (**B**) si-CDK1 and pcDNA3.1/iASPP were co-transfected into HCT116 and LoVo cells; the protein levels of p53, p21, Fas, Pro-caspase 8 and Cleaved caspase 8 were determined using Western blot assays. The data are presented as mean ± SD of three independent experiments. *P < 0.05, **P < 0.01 (compared to control group), ^##^*P* < 0.01 (compared to si-CDK1 + pcDNA3.1 group.).

The effect of cyclin B1/CDK1 on iASPP phosphorylation has been demonstrated [15]; moreover, in HT29 cells, ethanol extract of *S. rufopilosa* induced cell cycle arrest at G2/M through regulation of cyclin B, CDK1 and CDK inhibitor p21. In addition to the indicated factors, p53, Fas, a transmembrane protein that belongs to the family of tumor necrosis factor receptor superfamily, which binds to FasL to initiate apoptosis of apoptotic signaling, Bax, a pro-apoptotic protein, and caspase 3, 8, and 9 have been reported to be involved in the degradation of PARP [16]. According to these previous studies, we speculated that p53 pathway and related factors were involved in CRC cell proliferation regulation. To verify this speculation, we monitored the protein levels of p53, CDK inhibitor p21, death receptor Fas, caspase 8 (Pro-caspase 8 and activated Cleaved-caspase 8) in si-CDK1 and pcDNA3.1/iASPP-co-transfected HCT116 and LoVo cells. Results from Western blot assays showed that iASPP overexpression reduced the protein levels of p53, p21, Fas and Cleaved-caspase 8, increased Pro-caspase 8 protein level; whereas CDK1 knockdown increased the protein levels of p53, p21, Fas and Cleaved-caspase 8, reduced Pro-caspase 8 protein level; the effect of iASPP on the indicated proteins could be partially reversed by CDK1 knockdown (Figure 5A and 5B). These data suggested that CDK1 interacted with iASPP to affect CRC cell proliferation and apoptosis, and this progression was p53-dependent.

### Effect of iASPP and CDK1 expression on prognosis of CRC

**Figure 6:**
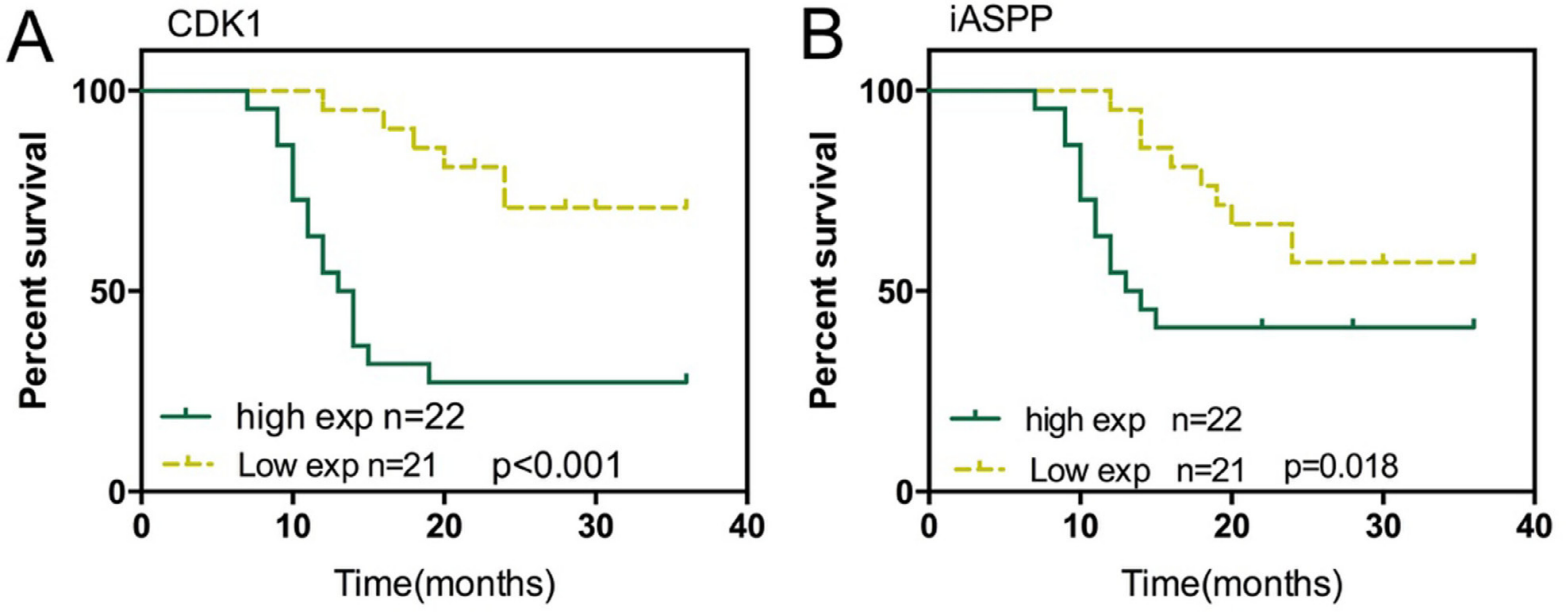
Effect of iASPP and CDK1 expression on prognosis of CRC (A) Kaplan-Meier overall survival curves for 43 patients with CRC classified according to relative CDK1 expression level (**B**) Kaplan-Meier overall survival curves for 43 patients with CRC classified according to relative iASPP expression level.

As we revealed, CDK1 and iASPP was highly expressed in CRC tissues, and higher expressed in tissued derived from the patients in advanced stages; here we further evaluated the effect of iASPP and CDK1 expression on prognosis of CRC. 43 cases of CRC tissues were divided into two groups: a high CDK1 expression group (above the median CDK1 expression, *n* = 22) and a low CDK1 expression group (below the median CDK1 expression, *n* = 21). To determine the potential relationship between CDK1 expression and the patients’ prognosis, Kaplan-Meier analysis and log-rank test were used to evaluate the effects of CDK1 expression on survival. The results indicated that patients with higher CDK1 expression had a significantly poorer prognosis compared to patients with lower CDK1 expression (*P* < 0.001) (Figure 6A). Similarly, the potential relationship between iASPP expression and the patients’ prognosis was also analyzed. Kaplan-Meier analysis and log-rank test exhibited that patients with higher iASPP expression (above the median iASPP expression, *n* = 22) had a significantly poorer prognosis compared to patients with lower iASPP expression (below the median CDK1 expression, *n* = 21, *P* = 0.018). These data suggested that CDK1 and iASPP was correlated with poorer CRC prognosis, respectively; targeting CDK1 and iASPP to inhibit their expression, thus to rescue the expression of p53 pathway-related factors showed to be a promising strategy in CRC treatment.

## DISCUSSION

CDK1, also known as cell division cycle protein 2 homolog, is a highly conserved protein that functions as a serine/threonine kinase, and is a key player in G1/S and G2/M phase transitions during cell proliferation through promoteing the M-phase process [6, 17]. Cyclin B-CDK1 is involved in cell survival during the mitotic checkpoint [18]. In CDK1 highly-expressed cancer cells, a higher cell proliferation capability is observed, which is might partially responsible for pooror clinical outcomes in patients with this type of tumor [5]. Thus, targeting CDK1 to efficiently suppress its expression could induce cell cycle arrest at the G2-M phase, thus to prevent the mitosis of cancer cell, finally cause increased apoptosis of cancer cell [18, 19]. Due to its higher expression in cancer cells, CDK1 might serve as not only a therapeutic target but also a prognosis marker.

In addition to CDK1, iASPP is also closely related to cell proliferation. Silencing of iASPP could result in suppressed U251 cell growth and G0/G1 cell cycle arrest through regulation of CDK inhibitor p21Waf1 [11]. After knockdown of iASPP in hepatocellular carcinoma cells, the proliferation of cancer cell was suppressed, tumor growth inhibited [20]. Moreover, it has also been reported that iASPP expression in colorectal cancer tissues was higher than that of the adjacent normal tissues [21]; through targeting iASPP, miR-124 exerts inhibitory effect on CRC cell viability, DNA synthesis capability, and colony formation [21].

Here, we monitored the mRNA expression of these two essential factors, CDK1 and iASPP in the CRC tissues derived from patients at different stages. Consistent with previous studies, the mRNA expression of both CDK1 and iASPP was significantly up-regulated in CRC tissues, compared to the matched adjacent normal tissues, and much higher in the CRC tissues of advanced stages (stage III and stage IV). Through the Spearman's rank correlation analysis of CDK1 and iASPP mRNA expression in 43 paired CRC and normal tissues, we found that CDK1 and iASPP mRNA expression was positively correlated with each other. As we mentioned, Lu et al. [15] reported cyclin B1/CDK1 phosphorylation of iASPP, finally caused increased p53 inhibition. Here we also found out that iASPP protein might interact with up to 20 proteins, including CDK1, through Cytoscape. These data suggested that CDK1 and iASPP protein might interact with each other in CRC.

To verify this interaction, we monitored the protein levels of CDK1 and iASPP in five CRC cell lines; as exhibited by Western blot assays, CDK1 protein and iASPP protein was increased in CRC cell lines, and positively correlated with each other. These prompted us to further confirm the interaction between CDK1 protein and iASPP protein in CRC cell lines, and their functional roles. As exhibited by GST pull down and Co-IP assays, iASPP could be captured and detected by Western blot in the precipitated product for CDK1, proving the interaction between CDK1 protein and iASPP protein. We also generated a binding structure schematic diagram of CDK1 interacting with iASPP using PyMol, which showed two possible independent interacting regions between CDK1 protein and iASPP protein, Tyr174 and Tyr673.

After confirming the interaction between CDK1 protein and iASPP protein, we further evaluated their functional roles in CRC cell proliferation and apoptosis. Commonly, the high expression of CDK1 is associated with over-proliferation and can be a prognostic factor in many cancers, including epithelial ovarian cancer [22], hepatocellular carcinoma [23], non-Hodgkin's lymphomas [24] and prostate cancer [25]. In addition to CDK1, iASPP has also been reported to be related to cancer cell proliferation [20, 21, 26, 27]. Here we achieved CDK1 knockdown and forced iASPP expression to evaluate the detailed function of CDK1 and iASPP interaction. After forced iASPP expression, CRC cell proliferation showed to be promoted, apoptosis repressed; whereas after CDK1 knockdown, CRC cell proliferation showed to be repressed, apoptosis promoted. Moreover, the effect of iASPP on CRC cell proliferation and apoptosis could be partially reversed by CDK1 knockdown. These data indicated that CDK1 protein interacted with iASPP protein to affect CRC cell proliferation and apoptosis. How do they exert their functions in CRC cell proliferation regulation?

According to previous studies, iASPP inhibits cell apoptosis and promotes cell proliferation through inhibiting p53-dependent apoptosis pathway [15, 28, 29]. In CRC cell line HT29, ethanol extract of *S. rufopilosa* induced cell cycle arrest at G2/M through regulation of cyclin B, CDK1 and CDK inhibitor p21. In addition to the indicated factors, p53, Fas, a transmembrane protein that belongs to the family of tumor necrosis factor receptor superfamily, which binds to FasL to initiate apoptosis of apoptotic signaling, Bax, a pro-apoptotic protein, and caspase 3, 8, and 9 have been reported to be involved in the degradation of PARP [16]. p53, a transcription factor which regulates expression of many genes in normal cells, has also been reported to mediate cell cycle arrest, senescence and/or apoptosis in several cancers [30–32]. In addition to its direct effect on inducing cell apoptosis, p53 is also able to indirectly mediate repression on cell proliferation by sequestering transcriptional activators including CDKN1A/p21, and then subsequently downregulate E2Fs activity [33, 34]. Is p53 pathway involved in CRC cell proliferation regulation? We further investigated this possibility. In si-CDK1 and pcDNA3.1/iASPP-co-transfected HCT116 and LoVo cells, the protein levels of p53 pathway-related factors: p53, CDK inhibitor p21, death receptor Fas and activated Cleaved-caspase 8 showed to be reduced by forced iASPP expression, increased by CDK1 knockdown; whereas Pro-caspase 8 protein was increased by CDK1 knockdown, reduced by forced iASPP expression. In addition, the effect of iASPP on the indicated proteins could be partially reversed by CDK1 knockdown, indicating that CDK1 and iASPP interaction regulated CRC cell proliferation and apoptosis through p53 pathway.

Finally, we evaluated the prognositic effects of CDK1 and iASPP in CRC. Through performing Kaplan-Meier analysis and log-rank test, we found that high CDK1 or iASPP expression was correlated with shorter survival percentage, respectively. Since we have revealed that the expression of CDK1 and iASPP at mRNA and protein levels were both increased in tumor tissues; suppressing CDK1 and iASPP expression in tumor tissues present a promising strategy for CRC treatment. In recent years, non-coding RNAs have been frequently reported to play an essential role in cell physiological activity in cancers, including cell proliferation, migration and apoptosis through regulation of related factors [35–37]. To search for the efficient upstream inhibitor of CDK1 and iASPP, thus to downregulate CDK1 and iASPP expression from the aspect of non-coding RNAs will become a great potential for research.

Taken together, we revealed that CDK1 and iASPP was up-regulated in CRC tissues and cell lines; CDK1 protein interacted with iASPP protein to affect CRC cell proliferation and apoptosis through p53 apoptosis pathway. CDK1 and iASPP might serve as not only promising targets in CRC treatment, but also efficient prognostic markers.

## MATERIALS AND METHODS

### Tissues, cell lines and transfection

A large panel of 43 paired CRC tissues and matched adjacent normal tissues were obtained from patients who received treatment at the Second Xiangya Hospital, Central South University (Changsha, China) under the approval of the Ethic Committee of the Second Xiangya Hospital, Central South University. The tissue samples were snap-frozen in liquid nitrogen stored at −80°C.

Human CRC cell lines, HCT116, HCT28, LoVo, SW480 and SW620 were purchased from ATCC (Rockville, MD, USA), maintained in DMEM supplied with 5% FCS (Commonwealth Serum Laboratories, Melbourne, VIC, Australia). CRL-1831, a normal human colon epithelial cell line, was obtained from ATCC, cultured in a 1:1 mixture of Ham's F12 and modified DMEM containing HEPES (25 mM), cholera toxin (10 ng/ml), insulin (5 mg/ml), transferrin (5 mg/ml) and hydrocortisone (100 ng/ml) (Sigma-Aldrich, Castle Hill, NSW, Australia).

A si-CDK1 vector was used to achieve knockdown of CDK1 (GeneCopoecia, Guangzhou, China). A pcDNA3.1/iASPP vector was used to achieve iASPP overexpression (GeneCopoecia, Guangzhou, China). Cells were plated in 6-well plates or 96-well plates, transfected, incubated for 24 h or 48 h and used for further assays or RNA/protein extraction.

### RNA extraction and SYBR green quantitative PCR analysis

We extracted total RNA from cells using Trizol reagent (Invitrogen, CA, USA). CDK1 mRNA and iASPP mRNA expression was measured by the SYBR green qPCR assay (Takara, Dalian, China). The data were processed using the 2^−ΔΔCT^ method.

### MTT assay

After seeding 2 × 10^3^ transfected cells/well into 96-well culture plates we assessed the viability of HCT116 and LoVo cells at six time points (12, 24, 36, 48, 60, 72 h). In brief, quantification of mitochondrial dehydrogenase activity was achieved through the enzymatic conversion of MTT [3-(4,5-dimethyldiazol-2-yl)-2,5- diphenyltetrazolium bromide; Sigma-Aldrich, MO, USA] to a colored formazan product. MTT (10 μl, 10 mg/ml) was added to the cells, incubated for 4 h, and we terminated the reaction by removal of the supernatant and add of 100 μl DMSO to dissolve the formazan product. After 0.5 h, the optical density (OD) of each well was measured at 570 nm using a plate reader (ELx808 Bio-Tek Instruments, City, ST, USA).

### Flow cytometer assay

For apoptosis analysis, quantification of apoptotic cells was performed with Annexin V-FITC apoptosis detection kit (Keygen, China). Briefly, the cell samples were harvested with 0.25% trypsin without EDTA after 48 hours of infection and then washed twice with ice-cold PBS and re-suspended in 500 μl binding buffer. Then cells were incubated with 5 μl Annexin V-FITC specific antibodies and 5 μl propidium iodide (PI) then incubated for 15–20 minutes in dark and detected by BD Accuri C6 flow cytometer (BD, USA) with the excitation wavelength of Ex = 488 nm and emission wavelength of Em = 530 nm. Each experiment was repeated three times in triplicate.

### Western blot analysis

The protein contents of CDK1, iASPP, p53, p21, Fas, Pro-caspase8 and Cleaved caspase8 in CRC cells was detected by performing Immunoblotting. We lysed cultured or transfected cells in RIPA buffer with 1% PMSF and loaded protein onto an SDS-PAGE minigel and transferred them onto PVDF membrane. After probed with the following antibodies: CDK1 (Cat# A17, Abcam, MA, USA), iASPP (ab34898, Abcam), p53 (Cat# PAb 240, Abcam), p21 (Cat# EPR362, Abcam), Fas (Cat# EPR5700, Abcam), Pro-caspase8 (C7849, Sigma-Aldrich, USA) and Cleaved caspase8 (Cat# E7, Abcam) at 4°C overnight, the blots were then incubated with HRP-conjugated secondary antibody (1:5000). Enhanced chemiluminescence (ECL) Substrates were used to visualize signals (Millipore, MA, USA). GAPDH was used as an endogenous protein for normalization.

### Prediction of interaction between CDK1 and iASPP

A website of structural biology of small peptides and protein structures, PEP-FOLD3 was used to predict the interaction between CDK1 protein and iASPP (http://bioserv.rpbs.univ-paris-diderot.fr/services/PEP-FOLD3).

### Gene cloning and plasmid construction for GST pull-down and Co-Immunoprecipitation

All genes were amplified from Human cDNA by PCR. For GST-pull down, the sequence encoding CDK1 was cloned into the pGEX-6P-1 vector which contained opening reading frame (ORF) of GST tag at the N-terminus (GST-CDK1). The gene encoding iASPP was cloned into pET22b (+) vector which contained ORF of 6 × His tag (His-iASPP). Meanwhile, For Co-IP assay, the sequence encoding CDK1 and iASPP were cloned into the pcDNA-Flag or pcDNA-Myc vector, named pcDNA-Flag-CDK1 and pcDNA-Myc-iASPP, respectively.

### Recombinant protein expression and purification

GST-CDK1 and His-iASPP were transformed into Escherichia coli BL21 (DE3), respectively. GST-CDK1- and/or His-iASPP-transformed cells were maitained in Luria-Bertani (LB) at 37°C till OD_600_ = 0.8, and then 0.5 mM IPTG was added. Cell cultures were maintained at 37°C for 5 hours for protein expression. Cells were then collected through 14000 g centrifugation, and then being resuspended in 20 mL lysis buffer (1% Triton X-100, 1 mM PMSF, 1×PBS, pH 7.6), lysed by sonication. Precipitate was then removed from the lysate through 14000 g centrifugation and kept at 4°C for 20 min. His-iASPP supernatant was loaded on Ni^2+^-NTA column (GE Health, chelating sepharose, 17-0575-02) balanced with 20 mL lysis buffer and washed with 20 mL lysis buffer containing 50 mM imidazol. GST-CDK1 supernatant was loaded on GSTrap FF colum (Amersham Biosciences, USA) balanced with 20 mL lysis buffer and washed with 20 mL lysis buffer containing 20 mM Tris-HCl, 20 mM GSH, 1mM DDT and 1 mM EDTA. The recombinant protein were then collected and dialyzed against reaction buffer containing 20 mM Tris, 100 mM NaCl, 1 mM DTT and 1 mM EDTA at 4°C.

### GST pull-down assay

GST-fused proteins were purified according to the earlier described method and incubated with glutathione sepharose beads (GE Health, Glutathione Sepharose 4B, 17-0756-01) at 37°C for 30 minutes. The beads were then collected and washed 3 times. 0.1 mg/mL of input proteins were dissolved in the reaction buffer (20 mM Tris, 100 mM NaCl, 1 mM DTT and 1 mM EDTA) and incubated with the beads at 37°C for 30 minutes. The supernatant was removed, the beads were then washed with the reaction buffer 4 times. The target proteins were collected after washed down with 10% SDS. These elutes were then analyzed and detected using Western blotting assays.

### Co-Immunoprecipitation (Co-IP) assay

The eukaryotic expression vectors, pcDNA-Flag-CDK1 and pcDNA-Myc-iASPP, which express CDK1 and iASPP, respectively, were constructed and co-transfected into HCT116 and LoVo cells. Empty vectors were co-transfected into target cells as controls. 36 h after transfection, the cells were treated and the proteins were extracted. Flag monoclonal antibodies were used for IP testing, followed by Western blot detection using Flag and Myc antibodies. In order to exclude the effect of DNase and RNase, cell lysates were treated with 5 mg/ml Dnase and Rnase, respectively.

### Statistical analysis

Data from at least three independent experiments were processed using SPSS 17.0 statistical software (SPSS, Chicago, IL, USA) and exhibited as mean ± SD. The differences between groups were compared by using Student's *t* test. The differences among more than two groups were evaluated using the one-way ANOVA. A *P* value of < 0.05 was considered statistically significant.
